# Crystal structure of 4α-hy­droxy-5α,8β(H)-eudesm-7(11)-en-8,12-olide monohydrate

**DOI:** 10.1107/S2056989015011251

**Published:** 2015-06-27

**Authors:** Qiang-Qiang Lu, Xin-Wei Shi, Xing-Ke Yang

**Affiliations:** a Xi’an Botanical Garden, Institute of Botany of Shaanxi Province, Xi’an 710061, People’s Republic of China; b Shaanxi Province Academy of Sciences, Xi’an 710061, People’s Republic of China

**Keywords:** crystal structure, eudesmane sesquiterpenoid, hydrogen bonds, *Chloranthus japonicus*

## Abstract

The title compound, C_15_H_22_O_3_·H_2_O, is a natural producr isolated from *Chloranthus japonicus*, which is a eudesmane sesquiterpenoid. The two *trans*-fused six-membered rings have chair confomations. In the crystal, O—H⋯O hydrogen bonds link the components into corrugated layers parallel to the *bc* plane. There are C—H⋯O inter­actions present within and between the layers.

## Related literature   

For the products isolated from the genus *Chloranthus*, see: Xiao *et al.* (2010 ▸); Sun *et al.* (2012 ▸). For the crystal structure of the related compound 6β-hy­droxy­eremophil-7(11)-en-8β,12-olide, see: Su *et al.* (2011 ▸).
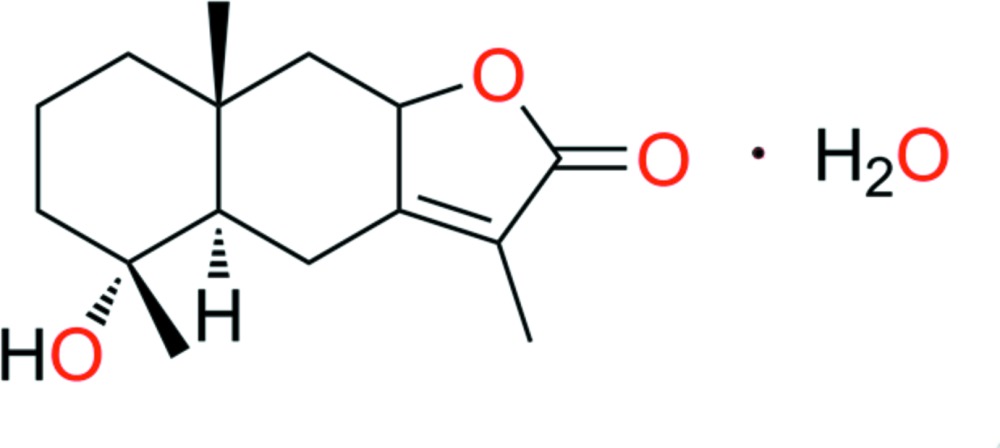


## Experimental   

### Crystal data  

C_15_H_22_O_3_·H_2_O
*M* *_r_* = 268.34
Monoclinic, 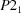

*a* = 10.2495 (2) Å
*b* = 7.1061 (1) Å
*c* = 10.5275 (2) Å
β = 100.026 (1)°
*V* = 755.05 (2) Å^3^
*Z* = 2
Cu *K*α radiation
μ = 0.68 mm^−1^
*T* = 298 K
0.40 × 0.40 × 0.30 mm

### Data collection  

Bruker SMART CCD area-detector diffractometer
Absorption correction: multi-scan (*SADABS*; Bruker, 2002 ▸) *T*_min_ = 0.772, *T*_max_ = 0.821
3081 measured reflections
1339 independent reflections
1303 reflections with *I* > 2σ(*I*)
*R*_int_ = 0.018

### Refinement  

*R*[*F* ^2^ > 2σ(*F* ^2^)] = 0.054
*wR*(*F* ^2^) = 0.130
*S* = 1.14
1339 reflections
174 parameters
1 restraint
H-atom parameters constrained
Δρ_max_ = 0.22 e Å^−3^
Δρ_min_ = −0.34 e Å^−3^

### 

Data collection: *SMART* (Bruker, 2002 ▸); cell refinement: *SAINT* (Bruker, 2002 ▸); data reduction: *SAINT*; program(s) used to solve structure: *SHELXS97* (Sheldrick, 2008 ▸); program(s) used to refine structure: *SHELXL97* (Sheldrick, 2008 ▸); molecular graphics: *SHELXTL*; software used to prepare material for publication: *ORTEP* (Johnson & Burnett, 1996 ▸).

## Supplementary Material

Crystal structure: contains datablock(s) I, New_Global_Publ_Block. DOI: 10.1107/S2056989015011251/cv5488sup1.cif

Structure factors: contains datablock(s) I. DOI: 10.1107/S2056989015011251/cv5488Isup2.hkl

Supporting information file. DOI: 10.1107/S2056989015011251/cv5488Isup4.cdx

**Fig. 1.**
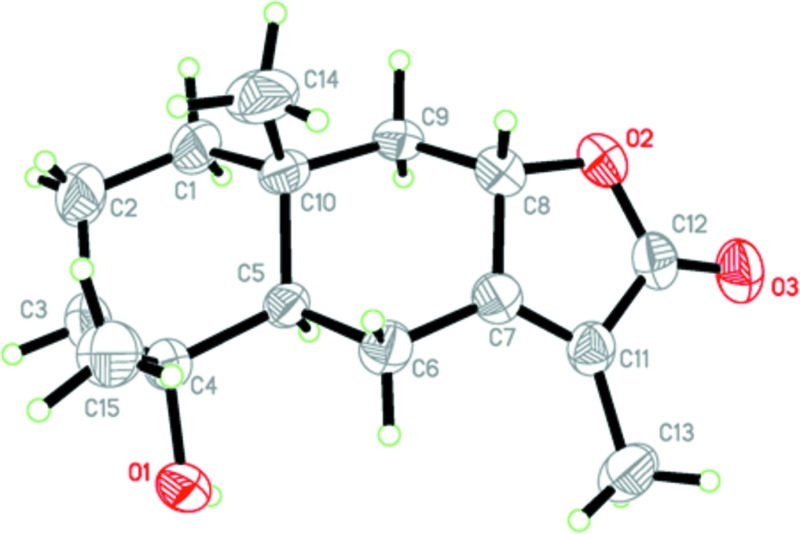
Molecular structure of the title compound showing the atomic numbering and 40% probability displacement ellipsoids.

. DOI: 10.1107/S2056989015011251/cv5488fig1.tif
Mol­ecular structure of the title compound showing the atomic numbering and 40% probability displacement ellipsoids.

**Fig. 2.**
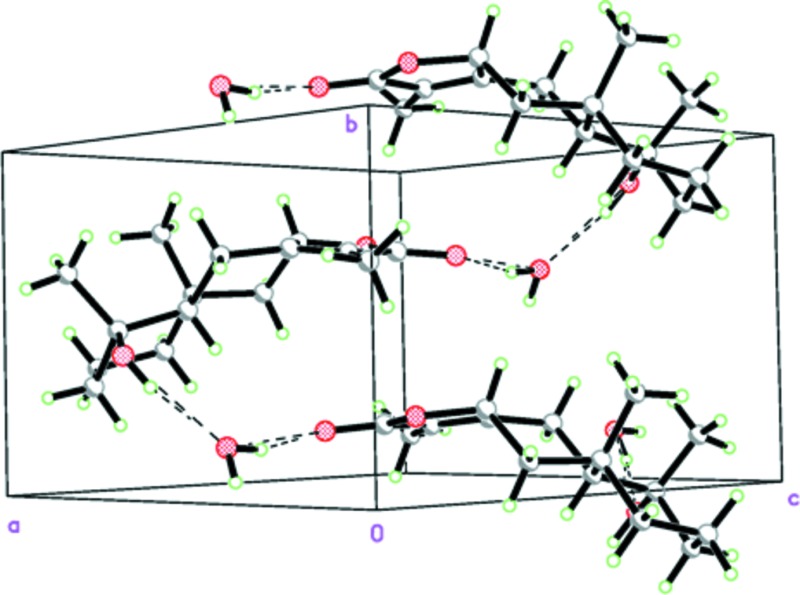
A portion of the crystal packing showing O—H···O hydrogen bonds as dashed lines.

. DOI: 10.1107/S2056989015011251/cv5488fig2.tif
A portion of the crystal packing showing O—H⋯O hydrogen bonds as dashed lines.

CCDC reference: 1405865

Additional supporting information:  crystallographic information; 3D view; checkCIF report

## Figures and Tables

**Table 1:** Hydrogen-bond geometry (, )

*D*H*A*	*D*H	H*A*	*D* *A*	*D*H*A*
C5H5O4^i^	0.98	2.63	3.407(3)	136
C8H8O3^ii^	0.98	2.64	3.308(4)	126
O1H1O4^i^	0.82	1.91	2.718(3)	169
O4H4*WA*O3^ii^	0.83	2.05	2.850(3)	162
O4H4*WB*O1^iii^	0.86	1.94	2.764(3)	159

Symmetry codes: (i) 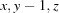; (ii) 
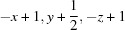
; (iii) 
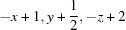
.

## supplementary crystallographic information

### S1. Comment

*Chloranthus japonicus* (Chloranthaceae,"yin-xian-cao"in chinese) is mainly distributed in the east of Asia and traditionally used as Chinese herbal medicine in the treatment of fractures, carbuncles, trauma, and rheumatism. In our current phytochemical investigation, the title compound - an eudesmane sesquiterpenoid, was isolated from the whole plant of *C. japonicus* for the first time.The compound was identified by NMR spectroscopic data, which were also elucidated by comparing with the literature data (Xiao *et al.*, 2010). Herein, we report its crystal structure.

The main molecule of the title compound consists of a fused three-ring system (Fig.1). The two methyl groups attached to C4 and C10 and the H atom at C8 are all in the axial position and assigned β-configuration, whereas, the hy­droxy group at C4 site has α-orientation.

In the crystal, inter­molecular O—H···O hydrogen bonds (Table 1, Fig. 2) link all moeties into corrugated layers parallel to *bc* plane, and weak C—H···O inter­actions (Table 1) consolidate further the crystal packing.

### S2. Experimental

The title compound was isolated from the whole plant of *C. japonicus* following the known procedure (Xiao *et al.*, 2010).

### S3. Refinement

The hydrogen atoms were placed in calculated positions and refined as riding with U_iso_(H) =1.2 U_eq_ (C) or 1.5 U_eq_(C, O). The positions of methyl and hy­droxy hydrogens were rotationally optimized.

### Crystal data

**Table:**

C_15_H_22_O_3_·H_2_O	*F*(000) = 292
*M**_r_* = 268.34	*D*_x_ = 1.180 Mg m^−^^3^
Monoclinic, *P*2_1_	Cu *K*α radiation, λ = 1.54178 Å
Hall symbol: P 2yb	Cell parameters from 2390 reflections
*a* = 10.2495 (2) Å	θ = 4.3–66.9°
*b* = 7.1061 (1) Å	µ = 0.68 mm^−^^1^
*c* = 10.5275 (2) Å	*T* = 298 K
β = 100.026 (1)°	Block, colourless
*V* = 755.05 (2) Å^3^	0.40 × 0.40 × 0.30 mm
*Z* = 2	

### Data collection

**Table:**

Bruker SMART CCD area-detector diffractometer	1339 independent reflections
Radiation source: fine-focus sealed tube	1303 reflections with *I* > 2σ(*I*)
Graphite monochromator	*R*_int_ = 0.018
phi and ω scans	θ_max_ = 64.0°, θ_min_ = 4.3°
Absorption correction: multi-scan (*SADABS*; Bruker, 2002)	*h* = −11→11
*T*_min_ = 0.772, *T*_max_ = 0.821	*k* = −6→8
3081 measured reflections	*l* = −11→12

### Refinement

**Table:**

Refinement on *F*^2^	Primary atom site location: structure-invariant direct methods
Least-squares matrix: full	Secondary atom site location: difference Fourier map
*R*[*F*^2^ > 2σ(*F*^2^)] = 0.054	Hydrogen site location: inferred from neighbouring sites
*wR*(*F*^2^) = 0.130	H-atom parameters constrained
*S* = 1.18	*w* = 1/[σ^2^(*F*_o_^2^) + (0.092*P*)^2^ + 0.043*P*] where *P* = (*F*_o_^2^ + 2*F*_c_^2^)/3
1339 reflections	(Δ/σ)_max_ < 0.001
174 parameters	Δρ_max_ = 0.22 e Å^−^^3^
1 restraint	Δρ_min_ = −0.34 e Å^−^^3^

### Special details

**Table:**

Geometry. All e.s.d.'s (except the e.s.d. in the dihedral angle between two l.s. planes) are estimated using the full covariance matrix. The cell e.s.d.'s are taken into account individually in the estimation of e.s.d.'s in distances, angles and torsion angles; correlations between e.s.d.'s in cell parameters are only used when they are defined by crystal symmetry. An approximate (isotropic) treatment of cell e.s.d.'s is used for estimating e.s.d.'s involving l.s. planes.
Refinement. Refinement of *F*^2^ against ALL reflections. The weighted *R*-factor *wR* and goodness of fit *S* are based on *F*^2^, conventional *R*-factors *R* are based on *F*, with *F* set to zero for negative *F*^2^. The threshold expression of *F*^2^ > σ(*F*^2^) is used only for calculating *R*-factors(gt) *etc*. and is not relevant to the choice of reflections for refinement. *R*-factors based on *F*^2^ are statistically about twice as large as those based on *F*, and *R*- factors based on ALL data will be even larger.

### Fractional atomic coordinates and isotropic or equivalent isotropic displacement parameters (Å^2^)

**Table:**

	*x*	*y*	*z*	*U*_iso_*/*U*_eq_	
C1	0.0506 (3)	−0.0853 (6)	0.6719 (3)	0.0694 (9)	
H1A	−0.0282	−0.0510	0.6111	0.083*	
H1B	0.0885	−0.1959	0.6384	0.083*	
C2	0.0108 (3)	−0.1346 (10)	0.7998 (3)	0.0938 (16)	
H2A	−0.0511	−0.2391	0.7881	0.113*	
H2B	−0.0331	−0.0277	0.8313	0.113*	
C3	0.1333 (3)	−0.1883 (7)	0.8991 (3)	0.0795 (12)	
H3A	0.1061	−0.2173	0.9807	0.095*	
H3B	0.1732	−0.3005	0.8700	0.095*	
C4	0.2366 (3)	−0.0303 (5)	0.9198 (2)	0.0545 (7)	
C5	0.2709 (2)	0.0271 (3)	0.7882 (2)	0.0400 (5)	
H5	0.3093	−0.0863	0.7567	0.048*	
C6	0.3814 (2)	0.1752 (4)	0.7989 (2)	0.0482 (6)	
H6A	0.3501	0.2943	0.8272	0.058*	
H6B	0.4571	0.1352	0.8616	0.058*	
C7	0.4201 (2)	0.1974 (3)	0.6697 (2)	0.0438 (5)	
C8	0.3125 (3)	0.2269 (4)	0.5558 (2)	0.0493 (6)	
H8	0.2754	0.3536	0.5590	0.059*	
C9	0.2037 (2)	0.0820 (4)	0.5517 (2)	0.0468 (6)	
H9A	0.2377	−0.0411	0.5347	0.056*	
H9B	0.1317	0.1120	0.4820	0.056*	
C10	0.1512 (2)	0.0772 (4)	0.6805 (2)	0.0480 (6)	
C11	0.5357 (2)	0.1772 (4)	0.6310 (2)	0.0469 (6)	
C12	0.5101 (3)	0.1860 (4)	0.4896 (3)	0.0507 (6)	
C13	0.6713 (3)	0.1422 (6)	0.7066 (3)	0.0659 (8)	
H13A	0.6695	0.1563	0.7970	0.099*	
H13B	0.7324	0.2312	0.6812	0.099*	
H13C	0.6990	0.0169	0.6901	0.099*	
C14	0.0832 (4)	0.2651 (6)	0.7002 (4)	0.0763 (10)	
H14A	0.0210	0.2956	0.6237	0.114*	
H14B	0.1487	0.3627	0.7171	0.114*	
H14C	0.0373	0.2545	0.7720	0.114*	
C15	0.1959 (3)	0.1322 (8)	0.9998 (3)	0.0838 (13)	
H15A	0.1939	0.0893	1.0859	0.126*	
H15B	0.1096	0.1764	0.9613	0.126*	
H15C	0.2588	0.2329	1.0025	0.126*	
O1	0.35464 (17)	−0.1021 (3)	0.99853 (14)	0.0547 (5)	
H1	0.3829	−0.1911	0.9619	0.082*	
O2	0.37912 (19)	0.2109 (3)	0.44542 (16)	0.0572 (5)	
O3	0.5884 (2)	0.1702 (4)	0.41479 (17)	0.0635 (6)	
O4	0.4640 (3)	0.6381 (4)	0.85924 (19)	0.0861 (8)	
H4WA	0.4612	0.6284	0.7803	0.103*	
H4WB	0.5174	0.5467	0.8853	0.103*	

### Atomic displacement parameters (Å^2^)

**Table:**

	*U*^11^	*U*^22^	*U*^33^	*U*^12^	*U*^13^	*U*^23^
C1	0.0398 (13)	0.106 (3)	0.0599 (16)	−0.0191 (17)	0.0010 (11)	0.0081 (18)
C2	0.0463 (14)	0.165 (5)	0.0689 (19)	−0.033 (2)	0.0068 (13)	0.020 (3)
C3	0.0572 (17)	0.123 (3)	0.0595 (16)	−0.025 (2)	0.0128 (13)	0.025 (2)
C4	0.0433 (13)	0.0820 (19)	0.0394 (12)	0.0026 (13)	0.0106 (9)	0.0026 (14)
C5	0.0330 (11)	0.0499 (13)	0.0371 (11)	0.0018 (9)	0.0065 (8)	−0.0020 (9)
C6	0.0447 (12)	0.0580 (14)	0.0414 (11)	−0.0076 (12)	0.0057 (9)	−0.0093 (12)
C7	0.0467 (12)	0.0426 (12)	0.0412 (11)	−0.0065 (10)	0.0054 (9)	−0.0028 (10)
C8	0.0525 (13)	0.0519 (13)	0.0432 (12)	0.0018 (11)	0.0072 (10)	0.0079 (11)
C9	0.0416 (12)	0.0578 (14)	0.0384 (11)	0.0031 (11)	−0.0006 (9)	0.0050 (11)
C10	0.0352 (11)	0.0638 (15)	0.0433 (12)	0.0049 (12)	0.0022 (9)	0.0014 (12)
C11	0.0474 (12)	0.0495 (13)	0.0441 (12)	−0.0106 (11)	0.0090 (9)	−0.0033 (11)
C12	0.0594 (14)	0.0468 (13)	0.0481 (12)	−0.0101 (12)	0.0152 (10)	0.0021 (12)
C13	0.0443 (13)	0.094 (2)	0.0591 (15)	−0.0076 (16)	0.0085 (11)	−0.0161 (17)
C14	0.0619 (18)	0.095 (3)	0.0706 (19)	0.0369 (19)	0.0087 (14)	0.0012 (18)
C15	0.0711 (19)	0.135 (4)	0.0500 (15)	0.036 (2)	0.0229 (13)	−0.010 (2)
O1	0.0528 (10)	0.0745 (13)	0.0355 (8)	0.0038 (9)	0.0039 (7)	0.0017 (8)
O2	0.0614 (11)	0.0679 (12)	0.0431 (9)	−0.0022 (10)	0.0112 (7)	0.0131 (9)
O3	0.0697 (12)	0.0720 (13)	0.0536 (10)	−0.0126 (11)	0.0248 (9)	0.0002 (11)
O4	0.1200 (19)	0.0856 (18)	0.0479 (10)	0.0426 (17)	0.0013 (11)	−0.0041 (11)

### Geometric parameters (Å, º)

**Table:**

C1—C2	1.515 (4)	C8—C9	1.512 (4)
C1—C10	1.541 (4)	C8—H8	0.9800
C1—H1A	0.9700	C9—C10	1.545 (3)
C1—H1B	0.9700	C9—H9A	0.9700
C2—C3	1.536 (5)	C9—H9B	0.9700
C2—H2A	0.9700	C10—C14	1.537 (4)
C2—H2B	0.9700	C11—C12	1.468 (4)
C3—C4	1.532 (5)	C11—C13	1.497 (4)
C3—H3A	0.9700	C12—O3	1.223 (3)
C3—H3B	0.9700	C12—O2	1.354 (4)
C4—O1	1.436 (3)	C13—H13A	0.9600
C4—C15	1.530 (5)	C13—H13B	0.9600
C4—C5	1.542 (3)	C13—H13C	0.9600
C5—C6	1.536 (3)	C14—H14A	0.9600
C5—C10	1.559 (3)	C14—H14B	0.9600
C5—H5	0.9800	C14—H14C	0.9600
C6—C7	1.490 (3)	C15—H15A	0.9600
C6—H6A	0.9700	C15—H15B	0.9600
C6—H6B	0.9700	C15—H15C	0.9600
C7—C11	1.325 (3)	O1—H1	0.8200
C7—C8	1.496 (3)	O4—H4WA	0.8291
C8—O2	1.451 (3)	O4—H4WB	0.8628
			
C2—C1—C10	113.7 (3)	O2—C8—H8	109.8
C2—C1—H1A	108.8	C7—C8—H8	109.8
C10—C1—H1A	108.8	C9—C8—H8	109.8
C2—C1—H1B	108.8	C8—C9—C10	110.9 (2)
C10—C1—H1B	108.8	C8—C9—H9A	109.5
H1A—C1—H1B	107.7	C10—C9—H9A	109.5
C1—C2—C3	110.4 (2)	C8—C9—H9B	109.5
C1—C2—H2A	109.6	C10—C9—H9B	109.5
C3—C2—H2A	109.6	H9A—C9—H9B	108.0
C1—C2—H2B	109.6	C14—C10—C1	110.2 (2)
C3—C2—H2B	109.6	C14—C10—C9	109.5 (2)
H2A—C2—H2B	108.1	C1—C10—C9	107.3 (2)
C4—C3—C2	112.2 (4)	C14—C10—C5	114.7 (2)
C4—C3—H3A	109.2	C1—C10—C5	107.8 (2)
C2—C3—H3A	109.2	C9—C10—C5	107.04 (18)
C4—C3—H3B	109.2	C7—C11—C12	107.2 (2)
C2—C3—H3B	109.2	C7—C11—C13	130.6 (2)
H3A—C3—H3B	107.9	C12—C11—C13	122.1 (2)
O1—C4—C15	103.5 (2)	O3—C12—O2	120.9 (3)
O1—C4—C3	108.3 (3)	O3—C12—C11	128.9 (3)
C15—C4—C3	112.5 (3)	O2—C12—C11	110.2 (2)
O1—C4—C5	108.18 (19)	C11—C13—H13A	109.4
C15—C4—C5	114.9 (3)	C11—C13—H13B	109.4
C3—C4—C5	109.1 (2)	H13A—C13—H13B	109.5
C6—C5—C4	113.3 (2)	C11—C13—H13C	109.5
C6—C5—C10	112.0 (2)	H13A—C13—H13C	109.5
C4—C5—C10	116.10 (19)	H13B—C13—H13C	109.5
C6—C5—H5	104.7	C10—C14—H14A	109.5
C4—C5—H5	104.7	C10—C14—H14B	109.5
C10—C5—H5	104.7	H14A—C14—H14B	109.5
C7—C6—C5	108.42 (18)	C10—C14—H14C	109.5
C7—C6—H6A	110.0	H14A—C14—H14C	109.5
C5—C6—H6A	110.0	H14B—C14—H14C	109.5
C7—C6—H6B	110.0	C4—C15—H15A	109.5
C5—C6—H6B	110.0	C4—C15—H15B	109.5
H6A—C6—H6B	108.4	H15A—C15—H15B	109.5
C11—C7—C6	131.6 (2)	C4—C15—H15C	109.5
C11—C7—C8	110.0 (2)	H15A—C15—H15C	109.5
C6—C7—C8	118.0 (2)	H15B—C15—H15C	109.5
O2—C8—C7	104.3 (2)	C4—O1—H1	109.5
O2—C8—C9	111.8 (2)	C12—O2—C8	108.18 (19)
C7—C8—C9	111.4 (2)	H4WA—O4—H4WB	99.5
			
C10—C1—C2—C3	−58.1 (6)	C2—C1—C10—C5	53.2 (4)
C1—C2—C3—C4	57.9 (5)	C8—C9—C10—C14	65.4 (3)
C2—C3—C4—O1	−171.6 (3)	C8—C9—C10—C1	−175.0 (2)
C2—C3—C4—C15	74.7 (3)	C8—C9—C10—C5	−59.5 (3)
C2—C3—C4—C5	−54.1 (4)	C6—C5—C10—C14	−60.4 (3)
O1—C4—C5—C6	−58.2 (3)	C4—C5—C10—C14	71.8 (3)
C15—C4—C5—C6	56.8 (3)	C6—C5—C10—C1	176.4 (2)
C3—C4—C5—C6	−175.8 (2)	C4—C5—C10—C1	−51.3 (3)
O1—C4—C5—C10	170.1 (2)	C6—C5—C10—C9	61.3 (3)
C15—C4—C5—C10	−74.9 (3)	C4—C5—C10—C9	−166.4 (2)
C3—C4—C5—C10	52.6 (3)	C6—C7—C11—C12	170.7 (3)
C4—C5—C6—C7	171.3 (2)	C8—C7—C11—C12	−1.8 (3)
C10—C5—C6—C7	−55.0 (3)	C6—C7—C11—C13	−6.6 (5)
C5—C6—C7—C11	−122.4 (3)	C8—C7—C11—C13	−179.0 (3)
C5—C6—C7—C8	49.6 (3)	C7—C11—C12—O3	−178.5 (3)
C11—C7—C8—O2	3.0 (3)	C13—C11—C12—O3	−1.0 (5)
C6—C7—C8—O2	−170.6 (2)	C7—C11—C12—O2	−0.1 (3)
C11—C7—C8—C9	123.7 (2)	C13—C11—C12—O2	177.4 (3)
C6—C7—C8—C9	−49.9 (3)	O3—C12—O2—C8	−179.4 (3)
O2—C8—C9—C10	169.80 (19)	C11—C12—O2—C8	2.1 (3)
C7—C8—C9—C10	53.6 (3)	C7—C8—O2—C12	−3.0 (3)
C2—C1—C10—C14	−72.7 (4)	C9—C8—O2—C12	−123.5 (2)
C2—C1—C10—C9	168.2 (3)		

### Hydrogen-bond geometry (Å, º)

**Table:**

*D*—H···*A*	*D*—H	H···*A*	*D*···*A*	*D*—H···*A*
C5—H5···O4^i^	0.98	2.63	3.407 (3)	136
C8—H8···O3^ii^	0.98	2.64	3.308 (4)	126
O1—H1···O4^i^	0.82	1.91	2.718 (3)	169
O4—H4*WA*···O3^ii^	0.83	2.05	2.850 (3)	162
O4—H4*WB*···O1^iii^	0.86	1.94	2.764 (3)	159

Symmetry codes: (i) *x*, *y*−1, *z*; (ii) −*x*+1, *y*+1/2, −*z*+1; (iii) −*x*+1, *y*+1/2, −*z*+2.
