# Supplementary figures and images for: Crystal structure of 4α-hy­droxy-5α,8β(H)-eudesm-7(11)-en-8,12-olide monohydrate
Source: Acta Crystallogr E Crystallogr Commun. 2015 Jun 27;71(Pt 7):o518. doi: 10.1107/S2056989015011251 (PMC4518932; doi:10.1107/S2056989015011251)

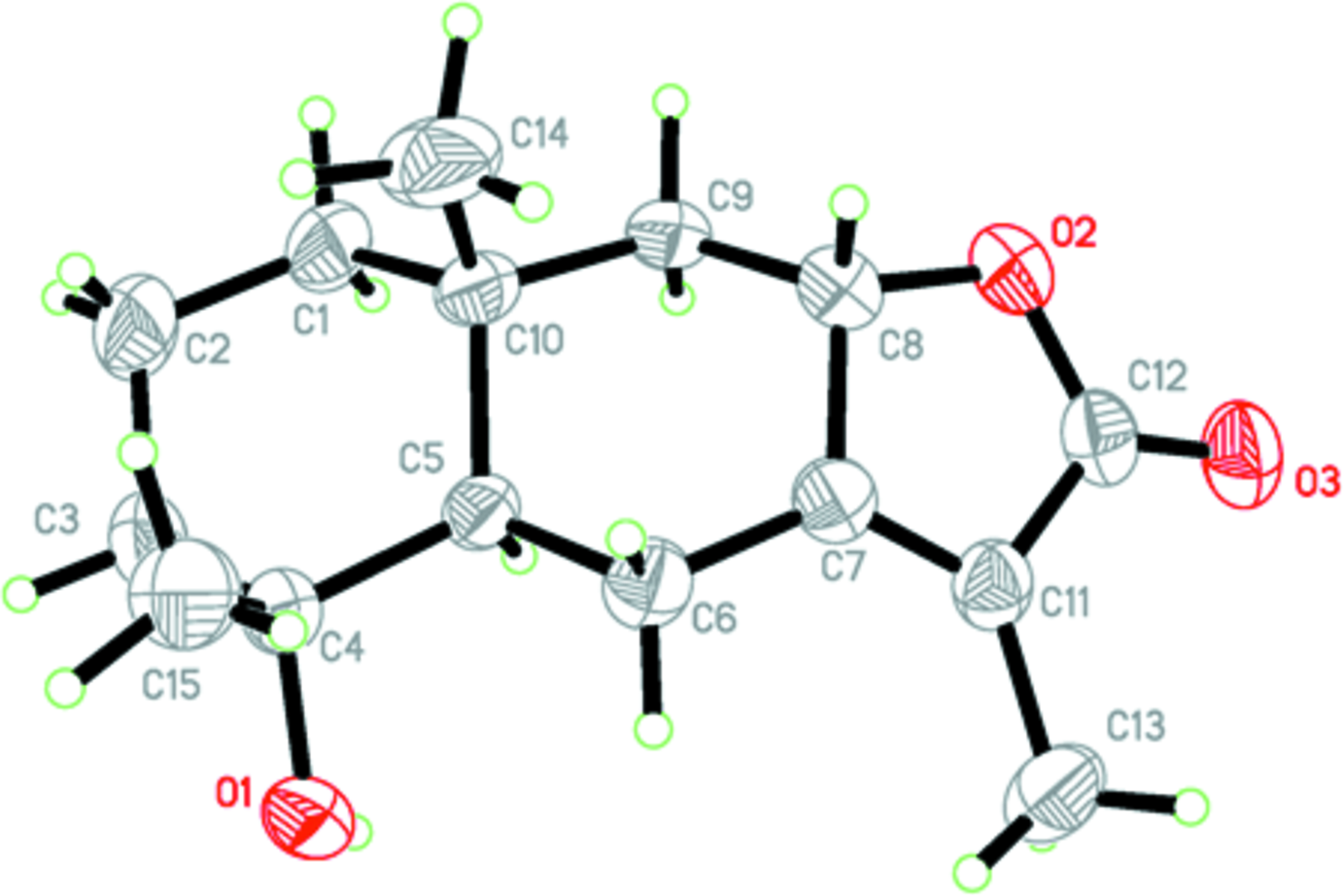

Supplement: Supplementary file 4 [file e-71-0o518-fig1.tif]

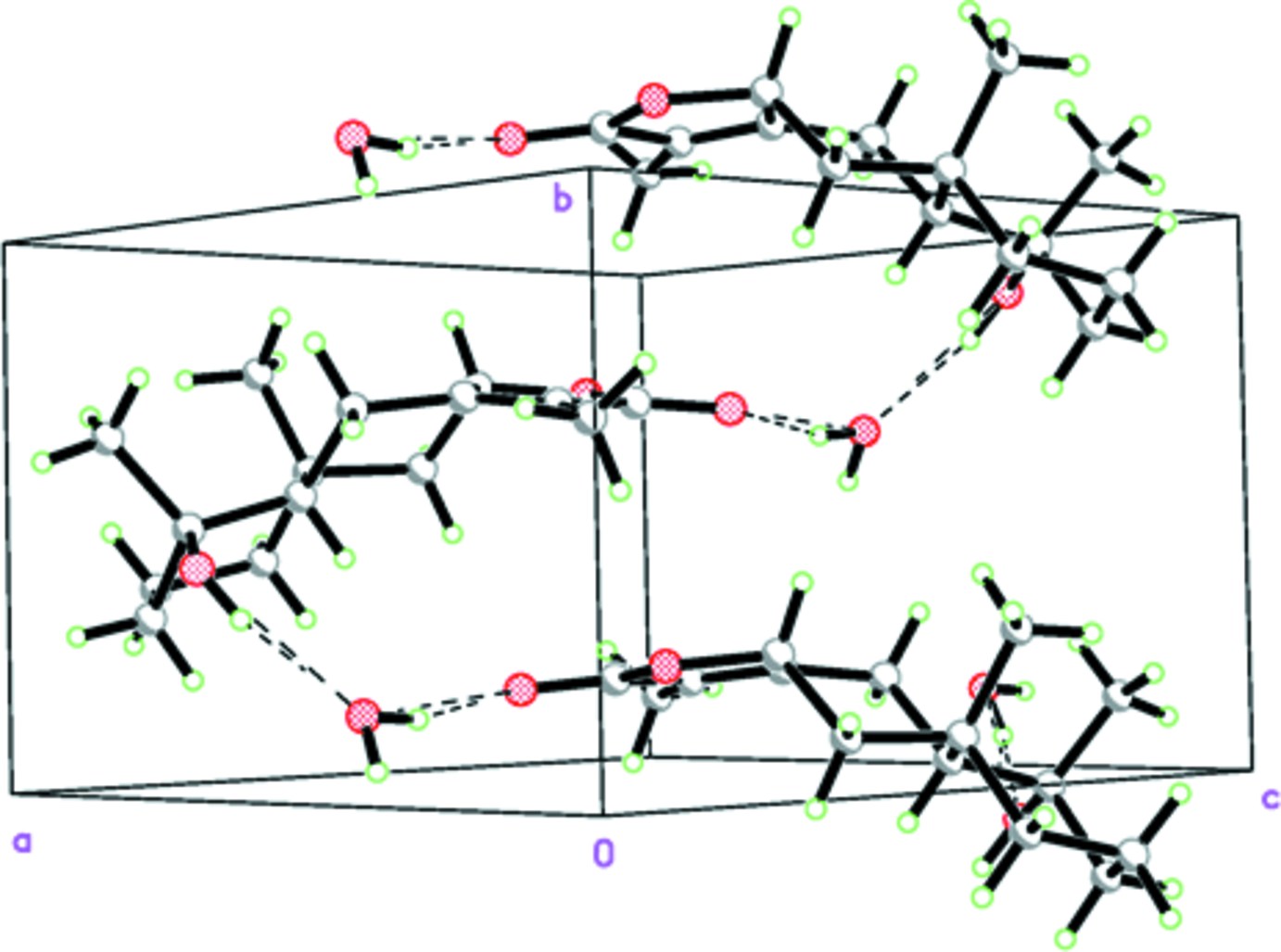

Supplement: Supplementary file 5 [file e-71-0o518-fig2.tif]
